# The multi-functional roles of menstrual blood-derived stem cells in regenerative medicine

**DOI:** 10.1186/s13287-018-1105-9

**Published:** 2019-01-03

**Authors:** Lijun Chen, Jingjing Qu, Charlie Xiang

**Affiliations:** 1 The Key Laboratory of Protein Chemistry and Developmental Biology of Ministry of Education, College of Life Sciences, Hunan Normal University, Changsha, 410081 China; 2 State Key Laboratory for Diagnosis and Treatment of Infectious Diseases, Collaborative Innovation Center for Diagnosis and Treatment of Infectious Diseases, The First Affiliated Hospital, School of Medicine, Zhejiang University, 79 Qingchun Road, Hangzhou, 310003 China; 3 Lung Cancer and Gastroenterology Department, Hunan Cancer Hospital, Affiliated Tumor Hospital of Xiangya Medical School of Central South University, Changsha, 410008 China

**Keywords:** Menstrual blood-derived stem cells, Cellular therapy, Adult stem cells, Menstrual blood, Regenerative medicine

## Abstract

Menstrual blood-derived stem cells (MenSCs) are a novel source of mesenchymal stem cells (MSCs). MenSCs are attracting more and more attention since their discovery in 2007. MenSCs also have no moral dilemma and show some unique features of known adult-derived stem cells, which provide an alternative source for the research and application in regenerative medicine. Currently, people are increasingly interested in their clinical potential due to their high proliferation, remarkable versatility, and periodic acquisition in a non-invasive manner with no other sources of MSCs that are comparable in adult tissue. In this review, the plasticity of pluripotent biological characteristics, immunophenotype and function, differentiative potential, and immunomodulatory properties are assessed. Furthermore, we also summarize their therapeutic effects and functional characteristics in various diseases, including liver disease, diabetes, stroke, Duchenne muscular dystrophy, ovarian-related disease, myocardial infarction, Asherman syndrome, Alzheimer’s disease, acute lung injury, cutaneous wound, endometriosis, and neurodegenerative diseases. Subsequently, the clinical potential of MenSCs is investigated. There is a need for a deeper understanding of its immunomodulatory and diagnostic properties with safety concern on a variety of environmental conditions (such as epidemiological backgrounds, age, hormonal status, and pre-contraceptive). In summary, MenSC has a great potential for reducing mortality and improving the quality of life of severe patients. As a kind of adult stem cells, MenSCs have multiple properties in treating a variety of diseases in regenerative medicine for future clinical applications.

## Background

Mesenchymal stem cells (MSCs), a heterogeneous subgroup of progenitor cells, have self-renewing capacity and differentiating potential into various specialized cell types, including osteoblasts, chondrocytes, and adipocytes [1]. MSC can be harvested from several adult tissues, such as bone marrow (BM), peripheral blood (PB), adipose tissue (AD), umbilical cord (UC), and placenta [2–6]. BM-MSCs have received the preferential attentions for exploring a variety of diseases in animal models and clinical trials [7–9]. Although BM-MSCs have obtained great priorities and predominant studies, the difficulty of separating BM-MSCs is a limiting factor owing to the requirements of invasive operation and ethical issue of donors [10, 11]. Therefore, exploring novel source of MSCs can be effectively used without these limitations.

Approximately a decade ago, Meng et al. and Cui et al. discovered a novel source of MSCs from human menstrual fluid, named menstrual blood-derived stem cells (MenSCs) [12, 13]. Throughout these years, more and more studies are focusing on MenSCs, a representative comparison was presented in six sources of MSCs, suggesting that MenSCs possessed higher proliferation rates and painless procedures, and almost no ethical issues [14]. In addition, some papers focus on the therapeutic potential and underlying mechanism of MenSCs. The purpose of this review is to provide an update based on current knowledge about MenSCs in their native environment, the basic characteristic, and a detailed description for therapeutic ways in a variety of diseases.

## Basic characteristic of MenSCs

### Definition and immunophenotype

At first, MenSCs are termed as endometrial regenerative cells [12, 15]. Subsequently, with advantages of non-invasive collection processes, high proliferation rate, pluripotency, and low immunogenicity, MenSCs were extensively studied [16–20]. Of course, there are some other names which were described by many researchers [21–37]. At present, the existing nomenclatures and detailed information of MenSCs are listed in Table 1. In this review, MenSCs are described with the uniform and convenient name throughout the paper.

**Table 1:** The existing nomenclatures for MenSCs in different literatures

Names	References
Endometrial regenerative cells	[12, 15]
Menstrual blood-derived stem cells	[16–20]
Menstrual-derived stem cells	[21]
Menstrual blood stem cells	[22]
Menstrual blood stromal stem cells	[23–25]
Menstrual stem cells	[26]
Menstrual blood-derived stromal stem cells	[27, 28]
Endometrial stem cells	[29]
Menstrual blood-derived endometrial stem cells	[30, 31]
Menstrual blood-derived mesenchymal stem cells	[32–34]
Menstrual blood progenitor cells	[35]
Endometrial mesenchymal stem cells	[36, 37]

In Table 2, we have completed a detailed comparison of the phenotypes published in the existing papers [12, 13, 16, 17, 19–21, 23, 26–28, 30–32, 34, 36–43]. According to Table 2, MenSCs were positive for several surface molecules, such as CD9, CD29, CD44, CD73, CD90, CD105, octamer binding transcription factor 4 (OCT-4), CD166, major histocompatibility complex I (MHC I), and C-X-C chemokine receptor type 4 (CXCR4). Among these molecules above, CD29, CD73, CD90, and CD105 were commonly identified for MSC markers. MenSCs also remained to have negative expressions for hematopoietic stem cell markers, such as CD34, CD45, and CD133. And CD14, CD38, and human leukocyte antigen-DR isotype (HLA-DR) were also negative. Interestingly, some papers were reported for the positive expression of embryonic markers and intracellular multipotent markers, such as OCT-4, c-kit proto-oncogene (c-kit)/CD117, and stage-specific embryonic antigen-4 (SSEA-4), which have not existed in MSCs from other sources. However, these findings were controversial, and some researchers showed that the expressions of c-kit and SSEA-4 were negative [12, 23, 26, 27, 42]. In our lab, we also performed these cells with surface molecule examination, and they were stable and consistent [19, 20, 34, 35, 38].

**Table 2:** Comparison of the different immunophenotypic profile of MenSCs

Markers	12	40	23	42	13	39	19, 20, 34, 38	16	27	26	17	36	43	28	32	37	21	41	30, 31
CD14	–				–					–			–		–			–	
CD34	–		–	–	–		–		–	–		–	–	–	–	–	–	–	–
CD38	–		–						–					–					
CD45	–		–	–	–	–	–		–	–	–	–	–	–		–	–	–	–
CD133	–		–		–	–			–						–		–	–	
STRO-1	–								–										
SSEA-4	–		+	+					–	–				–					+
Nanog	–	+																	+
CD9	+		+						+										
CD29	+		+	+	+		+	+	+			+	+		+	+			+
CD73	+				+		+	+		+	+			+	+		+	+	+
CD41a	+																		
CD44	+		+	+	+			+	+	+			+	+	+		+	+	+
CD90	+		+	+	+		+	+		+		+	+	+	+	+	+	+	+
CD105	+		+	+	+	+	+	+	+	+	+			+	+	+		+	+
OCT-4	+	+		+		+		+	+		+	+							
CXCR4		+	+																
CD166			+	+		+										+			
CD49f			+																
MHC I			+		+					+								+	+
MHC II (HLA-DR)			–		–		–			–		–			–			–	–
LIN			–																
CD117 (c-kit)			+	+	–		–			–	–	–				–			
CD13					+														
CD54					+														
CD55					+														
CD31					–					–									
CD50					–														
CD106						–													
Vimentin						+													
CD10								+	+										
CD271										–									
EpCAM										–									
CD49a										–							+		
SSEA-3										–									
TRA-1-60										–									
CD146											+						+		
CD40															–				
CD83															–				
CD86															–				
CD19																		–	
Sox2																		–	+
c-myc																		–	+

*STRO-1* stromal cell antigen 1, *LIN* lineage, *EpCAM* epithelial cell adhesion molecule, *TRA-1-60* tumor-rejection antigen-1-60, *Sox2* SRY (sex determining region Y)-box 2, *c-myc* c-myelocytomatosis

### Proliferation and differentiation

Studies by Meng et al. and our group have reported that MenSCs from young and healthy women could increase to one doubling every 20 h supplied with sufficient culture conditions, which was twice as fast as BM-MSCs (estimated 40–45 h) [12, 35]. MenSCs have similar phenotypes and properties compared with BM-MSCs, including spindles, classical three-line differentiation, and surface marker expression. A high rate of proliferation was contributed to the high expression of embryonic trophic factors and extracellular matrix (ECM) in MenSCs [44]. A high proliferative capacity is critical for future clinical research because cell-based treatment is usually dose-dependent along with cells from the lower passages; therefore, increasing the yield of the preliminary cells is necessary and considerable in clinical research. In addition, MenSCs have been extensively expanded in vitro and hardly showed obvious chromosomal abnormalities by our group and others [12, 23, 35]. Such a highly proliferating rate and stably genetic characteristic, as well as the apparent pluripotency, suggest that the novel stem cells may exhibit unexpected therapeutic properties.

MenSCs are also remarkable for their broad differentiation capacity. Currently, MenSCs can be induced as endothelial, cardiomyocytic, neurocytic, cartilaginous, myocytic, respiratory epithelial, pancreatic, hepatic, adipocytic, and osteogenic parts using appropriate differentiation techniques [12, 14, 26]. Hida et al. found that MenSCs exhibited cardiogenic differentiation in a scaffold culture system [45]. Lai’s team has confirmed that the differentiation of MenSCs into germ cells was induced in the appropriate medium [46]. Similarly, Liu et.al also proved that MenSCs had the capacity to differentiate into ovarian tissue-like cells [22]. Furthermore, our group and Khanjani et al. have shown that MenSCs could differentiate into functional hepatocyte-like cells by checking mature hepatocyte functions [17, 33, 39]. In addition, MenSCs had a potential for differentiation into glial lineages (neurosphere-like cells) by examining the expression of glial fibrillary acidic protein, oligosaccharide-2, and myelin basic protein [47].

### Immunomodulation properties

The extensive progress has been made in immunomodulatory properties for a multitude of inflammatory responses in inhibiting dendritic cells, B lymphocyte cells, T lymphocyte cells, mixed lymphocyte reaction (MLR), and promoting regulatory T cells (Tregs) [48]. However, the research on the immunomodulation of MenSCs is still in its infancy. It is worth noting that the endometrium is a part of the mucosal immune system, and MenSCs are extracted from menstrual blood, and their original sources are deciduous endometrial stem cells [44]. In fact, we only knew sporadic information of MenSC in animal disease models with autoimmune diseases [24, 25]. This emphasized the necessity for further research to assess immunosuppressive effects on immune-related molecules (such as MHC I, MHC II, CD40, and CD80/86) and other inflammatory-related cytokines (such as interferon-γ (IFN-γ), tumor necrosis factor α (TNF-α), interleukin-1 alpha (IL-1α), and interleukin-1 beta (IL-1β)) in MenSCs [49]. Therefore, immunoregulatory properties of MenSCs are currently unrecognized despite the unified management mechanism of MenSC-based therapy is explored in animal models and clinical researches.

## Practical application of MenSCs in tissue regeneration and disease therapy

At present, more and more registrations for a variety of diseases support the therapeutic benefits of MSC transplantation in clinical trials (www.clinicaltrials.gov). In contrast, the registrations of MenSCs are still few, and no more than 10 clinical trials are presented by searching “menstrual blood stem cells”. Actually, the therapeutic potential of MenSCs has already been recognized in several kinds of diseases in pre-clinical research, which is fundamental for future clinical applications in tissue repair and regenerative medicine. Similar to BM-MSCs, MenSCs also have several merits, including the ability to migrate into injury sites, differentiation into different cell lineages, secretion of soluble factors, and regulation of immune responses. Therefore, more researches need to be explored before MenSC becomes a common use in clinical application and treatment.

### Liver disease

Liver fibrosis is the universal phase of various chronic liver diseases and causes a huge public health issue due to high rates for the morbidity and mortality worldwide [50]. Fibrosis was a reversible process along with wound healing and characterized by accumulation of ECM protein at the site of an injured liver [51]. At present, orthotopic liver transplantation (OLT) is the most effective strategy for patients at the end stage of liver disease. However, due to lack of organ donors, surgical complications, lifelong immunosuppression, and high cost, its application has been limited to a large number of patients in the current condition. Recently, we have studied the therapeutic effect of MenSC transplantation in a mouse model of liver fibrosis induced by CCl_4_ (carbon tetrachloride) [20]. The results showed that MenSC had the effect on treating liver fibrosis. Liver function was improved via targeting activated hepatic stellate cells (HSCs), and collagen deposition was reduced after cell transplantation in liver fibrotic mice. Co-culture experiments showed that MenSCs restrained the proliferation of LX-2 cells (HSC line) through secretion of paracrine cytokines, including interleukin-6 (IL-6), IL-8, hepatocyte growth factor (HGF), monocyte chemoattractant protein 1 (MCP-1), growth-related oncogene (GRO), and osteoprotegerin (OPG). The results suggest that MenSC may be an attractive treatment for chronic liver disease by targeting HSCs via paracrine mediators.

Fulminant hepatic failure (FHF) is a life-threatening and sharply pathological reaction, which results in relatively high mortality by rapid necrosis of liver cells with the stimulation of a variety of acute injuries, such as hepatotoxic drugs, immune-mediated attacks, or viral infections [52]. The exosomes contain microRNA/lncRNA and adhesion molecules as well as small vesicles of secreted proteins, which mediate cellular signaling pathways both in vivo and in vitro [53]. Our group proved that MenSC-derived exosomes (MenSC-Ex) possessed therapeutic potential by inhibiting hepatocyte apoptosis in D-galactosamine (D-Gal) and lipopolysaccharide (LPS) induced FHF in mice, and we further showed that the levels of TNF-α, IL-6, and IL-1β were reduced by co-culture with AML12 hepatocytes (a normal mouse hepatocyte cell line) in vitro [19]. The study suggests that MenSC-Ex can improve liver function to increase the rate of survival in FHF model mice.

### Diabetes

Type 1 diabetes mellitus (T1DM), known as a kind of autoimmune diabetes, is a multifactorial disease by the deficiency of secreting insulin in islet β cells to influence the normal organism metabolism, ultimately leading to elevated blood glucose levels and a severe decline in insulin secretion [54]. Transplantation of human islets is currently the most effective treatment; due to the lack of pancreatic donors, it has been greatly restricted in the widespread application. Our team has studied the therapeutic effect of MenSCs and the underlying mechanism of β cell regeneration after MenSC transplantation in the T1DM mouse model [35]. From our study, MenSCs could facilitate β-cell regeneration and enhance the number of β cells by increasing the expressions of neurogenin 3 (ngn 3), forkhead box A2 (foxa 2), pancreatic and duodenal homeobox 1 (pdx 1), NK homeobox factor 6.1 (nkx 6.1), and paired box gene (pax) to activate endogenous progenitor cell differentiation post MenSC transplantation in T1DM mice. Clarifying the precise mechanism involved in MenSC-induced β-cell regeneration will facilitate the future use of MenSCs to treat diabetes.

### Ischemic stroke

Ischemic stroke, one of the leading causes of long-term disability, is a chain reaction of functional impairment that initially occurs during the identification phase of rapid physical and mental fluctuations [55]. Currently, ischemic stroke causes many patients producing permanent nerve damage, and stem cell therapy will help to improve and possibly restore the nerve function. Borlongan et al. demonstrated that MenSCs improved ischemic stroke in an oxygen glucose deprivation (OGD) rat model in vitro [40]. The behavioral and histological disorders were also significantly improved in the rat model of ischemic stroke by intracerebral/intravenous transplantation. Co-culture experiments showed that MenSCs significantly reduced cell death of OGD-exposed rat primary neurons through increasing vascular endothelial growth factor (VEGF), brain-derived neurotrophic factor (BDNF), and neurotrophin 3 (NT-3). The neurostructural and behavioral benefits afforded by transplanted MenSCs support their use as a kind of stem cell source for cell therapy in treating ischemic stroke.

### Duchenne muscular dystrophy

Duchenne muscular dystrophy (DMD) is a deadly x-linked muscle degeneration disease that consists of a potential genetic defect characterized by an enhanced inflammatory response [56]. DMD is an important part of the muscular dystrophy glycoprotein complex (DGC), which is involved in the relative stabilization of the sarcolemma and regulation of the interaction between the cytoskeleton and skeletal muscle and myocardial ECM. Umezawa’s team showed that MenSCs could restore muscle degeneration and repair skeletal muscle abnormalities by increasing muscle-like protein expression in immunodeficient DMD model mice [13]. In addition, MenSCs effectively differentiated into myoblasts/muscle cells after co-culture with mouse myoblast C2C12 in vitro, and these differentiated cells could express anti-atrophy muscle protein. It is suggested that MenSCs transform muscular dystrophic cells into anti-atrophic cells through trans-differentiation both in vitro and in vivo.

### Critical limb ischemia

Critical limb ischemia (CLI) refers to the final clinical stage along with the limb damage due to severe blood loss causing a series of pathological and physiological abnormalities that lead to limb pain or insufficient nutrition to support the legs [57]. Currently, although clinical trials have reported that autologous stem cells improve their symptoms by stimulating angiogenesis, the appropriate cell population of MSCs is still needed to explore. Murphy et al. demonstrated that administration of MenSCs improved CLI in a mouse model [58]. Although they did not explore the precise mechanism, they pointed out three possible reasons: (1) producing high levels of growth factors, IL-4, hypoxia inducible factor-1 alpha (HIF-1α), and matrix metalloproteinases (such as MMP3 and MMP10) with a paracrine role; (2) inhibiting the inflammatory response and blocking the pro-inflammatory signaling pathway; (3) producing a large amount of endothelial progenitor cells to mediate cell differentiation. Collectively, they suggest that MenSC represents a novel approach for treating the CLI by supplying an “off the shelf” therapeutic way, and it will provide a guideline for the feasibility of the proposed clinical trial in future.

### Ovarian-related disease

Ovarian cancer is one of the most deadly gynecological diseases for ambiguous symptoms and lack of reliable screening methods in many developed countries [59]. At present, platinum-based combination chemotherapy is the standard treatment for the past decade, but there is almost no improvement and progress. Cancer patients, especially women under the age of 40, are often suffering from reproductive damage related with premature ovarian failure (POF) and infertility in women. Lai et al. have demonstrated that MenSCs improved the estrous cycle and restored mouse fertility in the POF mouse model [36]. Wang et al. further explored that MenSCs could significantly improve the ovarian microenvironment by reducing granulosa cell apoptosis and ovarian interstitial fibrosis [21]. Transplanted MenSCs played an important role in ovarian function by secreting cytokines such as fibroblast growth factor 2 (FGF 2). MenSCs repair ovarian injury, improve ovarian function, and stimulate ovarian regeneration, which suggest that MenSCs may be a novel and effective strategy for the treatment of POF in regenerative medicine.

In addition, epithelial ovarian cancer (EOC) has been found to be advanced in most cases, with a combination of extensive abdominal metastasis, high recurrence, and chemoresistance [60]. Recently, Lai group found that MenSCs could improve the symptoms of EOC through tumor transplant animal model in vivo. Moreover, they further discovered that MenSCs induced angiogenic ability by inhibiting AKT/PKB (protein kinase B)-mediated degradation of the forkhead O-3a (FoxO3a) to induce cell cycle arrest, promote apoptosis, interfere with mitochondrial membrane function, and reduce EOC cells in co-culture models in vitro. These results suggest that MenSCs inhibit AKT-induced degradation of FoxO3a, which facilitates the anti-tumor properties of MenSCs on EOC in future regenerative medicine.

### Myocardial infarction

Myocardial infarction (MI), a type of coronary artery disease (CAD), is pathologically defined as the death of cardiomyocytes because of excessive ischemic condition [61]. Since MI has a long-term undiscovered phase, it can also be a major catastrophic event that causes sudden death or severe hemodynamic deterioration in patients. Hida et al. confirmed that the transplanted MenSCs significantly restored the damaged cardiac function in nude rat model [45]. In addition, Jiang et al. further demonstrated that MenSCs significantly reduced apoptosis, promoted cell proliferation, and recruited c-kit^+^ cells in an immunological MI model rats [29]. MenSCs could express some specific cytokines to activate AKT/extracellular signal-regulated kinases 1 and 2 (ERK 1/2)/signal transducers and activator of transcription 3 (STAT 3) and suppress p38 signaling pathway. Then Wang’s team found that MenSCs inhibited endothelial cell to mesenchymal transition (EMT), which helped to reduce the total number of cardiac fibroblasts and tissue fibrosis progression [62]. In addition, they verified that secreted exosomes of miR-21 mediated and enhanced the paracrine and cytoprotective effects through a transwell co-culture system in vitro. Exosomal microRNA (miR) array revealed that miR-21 targeted phosphatase and tensin homolog (PTEN) and the downstream of AKT [37]. These results suggest that MenSCs improve the damaged cardiac function in MI mainly through paracrine role and miRs deriving from exosome.

### Asherman syndrome

Asherman syndrome is caused by the formation of adhesions in the uterine cavity. Women with this disease often have many comprehensive and complicated symptoms, such as infertility, irregular menstruation (including amenorrhea, less menstruation, or dysmenorrhea), and repeated pregnancy loss [63]. Autologous MenSC transplantation significantly increased endometrial thickness (ET) in Asherman syndrome women in a total of 7 patients with Asherman syndrome in a non-controlled prospective and 3-year clinical study [28]. They showed that the ET of 5 women was significantly increased to 7 mm (a thickness to ensure embryo implantation). Four of these patients were subjected to frozen embryo transfer (FET). Surprisingly, one patient developed a spontaneous pregnancy only after the second MenSC transplant. This study suggests that autologous MenSC transplantation is a possible option for the treatment of Asherman syndrome in women.

### Alzheimer’s disease

Alzheimer’s disease, caused by amyloid-beta (Aβ) production, is progressive memory loss and cognitive dysfunction, and its neuropathological features are induced by the hyperphosphorylated tau proteins, which are composed of extracellular Aβ plaque deposits and intracellular neurofibrillary tangles (NTFs) [64]. Our group found that transplantation of MenSC in the brain of APP/PS1 mice could significantly improve the spatial learning characteristics and memory ability of AD in mouse model [34]. In addition, MenSCs significantly improved amyloid plaques in vivo and reduced tau hyperphosphorylation. It is worth noting that we also proved that intracranial transplantation of MenSCs significantly increased the expression of Aβ-degrading enzymes and decreased the level of pro-inflammatory cytokines to alter the microglia-associated phenotype. This result indicates that MenSCs can degrade Aβ and play an anti-inflammatory effect for improving AD in vivo.

### Acute lung injury

Acute lung injury (ALI) is a severe health burden worldwide due to its rapid attack and high mortality. Many factors can cause ALI, such as tidal volume, mechanical ventilation, or hypoxia; these injuries are often accompanied by inflammatory reactions, and once inflammatory reactions are sustained, the patients will face suffocation or even death [65]. Our group showed that MenSCs promoted the repair of injured lung by inhibiting the inflammatory response in LPS-induced ALI in mice [38]. Furthermore, MenSCs not only improved pulmonary microvascular permeability, reduced histopathological injury, and downregulated the expressions of IL-1β and caspase-3, but also upregulated the levels of IL-10, proliferating cell nuclear antigen (PCNA), and keratinocyte growth factor (KGF) in bronchoalveolar lavage fluid (BALF). MenSCs could also increase the survival rate of BEAS-2B cells (human normal lung epithelial cells) and inhibit LPS-induced cell apoptosis in a co-culture experiment. These results suggest that MenSC-based treatment may become an attractive strategy for improving ALI in regenerative medicine.

### Cutaneous wound

Cutaneous wound is repaired by coordinated biological progress to restore the original stage of damaged tissue, including cell proliferation and differentiation, and a variety of cell apoptosis, thereby producing multiple layers of connective tissue. The repaired skin is usually cured in the form of a scar, and the main purposes of the cutaneous regeneration are to understand how to induce skin to reconstruct damaged parts without forming scars [66]. Cuenca et al. revealed that MenSCs significantly improved wound healing and enhanced new blood vessel formation in a mouse excisional wound model [41]. They further discovered that MenSCs secreted some cytokines, including angiopoietin (Angpt), platelet-derived growth factor (PDGF), elastin (Eln), MMP3, and MMP10, allowing them to participate in wound repair. These results suggest that MenSCs promote wound healing and contribute to cutaneous regeneration.

### Endometriosis

Endometriosis is a common gynecological disorder defined as endometrial glands and interstitial growth outside the uterus, which is currently present in approximately 10% of women at reproductive age and 30% of infertile women [67]. Clinical interest and research are mainly focused on lesions and the directly affected diseases for the lack of understanding and exploration of the pathogenesis of women in different periods. Nikoo et al. found that MenSCs played a very important role by comparing the ability in morphology, CD marker expression, cell proliferation, invasion, adhesion, and some immunomodulatory molecules between women with endometriosis (E-MenSCs) and non-endometriosis (NE-MenSCs) [27]. In addition, the expressions of indoleamine 2,3-dioxygenase-1 (IDO1), cyclooxygenase-2 (COX-2), IFN-γ, IL-10, and MCP-1 were increased, and the level of forkhead transcription factor-3 (FOXP3) was reduced in co-culture of E-MenSCs and peripheral blood mononuclear cells (PBMCs) in vitro. These finding suggests that MenSC has a critical role in improving endometriosis.

## Clinical applications and safety concerns of MenSCs

Cellular therapies using MSCs are undergoing extensive preclinical and clinical trials. Especially, the progress of clinical trials of BM-MSCs is encouraging, a variety of diseases are researched in the I or II stage [68]. Compared to the most common BM-MSCs, the clinical trials of MSCs from other tissue sources (such as AD, PB, UC, and placenta, amniotic membrane, and menstrual blood) are a drop in the bucket. Bianco proposed that different human diseases could be reasonably used with different sources of MSCs, which was better to establish the physiological and pathological system to treat a variety of diseases for clinical applications [1]. Because MenSCs possess good immunosuppressive properties, they are able to intravenously inject large amounts of cells to injured body. From short-term studies, they are safe and reliable after cell transplantation, and they migrate into the inflammatory or injured sites, which has a regenerative inhibitory effect on inflammation [49]. Currently, no evidence of tumor or toxicity following administration of MenSC has been found in nude mice. Moreover, we assessed that MenSCs had significant inhibitory effects on tumor growth in a mouse glioma model [69]. No obvious physiological or serological abnormalities were observed in four patients with multiple sclerosis for the use of MenSCs [15]. Although researches indicate that MenSCs are rapidly evolving, it is not yet determined how long MenSCs can survive in foreign bodies and there are no data guaranteeing their long-term safety owing to lack of specific markers to monitor these cells in vivo [10, 68].

From a safety point of view, people are concerned with the collection procedure with standard process and isolated MenSCs are carried out under aseptic conditions in compliance with good manufacturing practice (GMP) release standards. At present, the high-quality and high-consistency of MenSCs are still scarce because there are no golden standardization and ideal molecular markers to verify them. The heterogeneity of MenSCs is derived from the variability of donors, different procedures of cell cultures, and a variety of environmental conditions (such as epidemiological backgrounds, age, hormonal status, and pre-contraceptive). These interventions may make MenSC transplantation of a routine clinical treatment and avoid unproven treatments that possess a health risk to the patients and may compromise the reputation of stem cell research and therapy.

## Future perspectives and conclusions

**Fig. 1:**
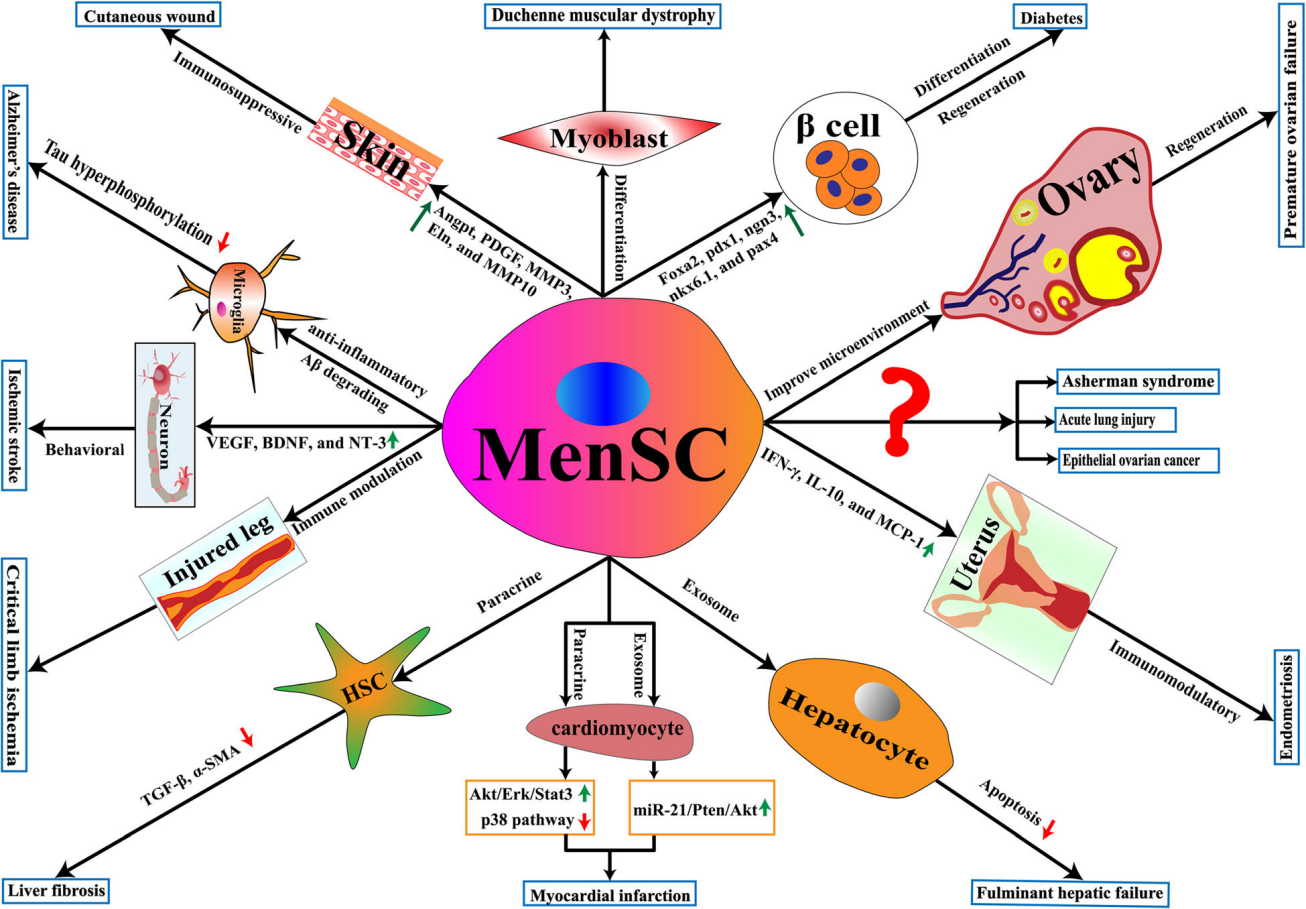
Schematic diagram of MenSCs in treating a diversity of diseases, including liver fibrosis, fulminant hepatic failure, diabetes, stroke, Duchenne muscular dystrophy, epithelial ovarian cancer, premature ovarian failure, myocardial infarction, Asherman syndrome, Alzheimer’s disease, acute lung injury, cutaneous wound, endometriosis, and neurodegenerative diseases. The blue boxes with characters show various diseases, the green arrow represents enhancement, and the red arrow represents decrement. Abbreviations: Erk, extracellular signal-regulated kinases; Stat 3, signal transducers and activator of transcription 3; Akt, PKB (protein kinase B); miR-21, microRNAs 21; Pten, phosphatase and tensin homolog; Aβ, amyloid-beta; IL-10, interleukin 10; α-SMA, α-smooth muscle actin; TGF-β, transforming growth factor-β; IFN-γ, interferon-γ; VEGF, vascular endothelial growth factor; BDNF, brain derived neurotrophic factor; NT-3, neurotrophin 3; Angpt, angiopoietin; PDGF, platelet-derived growth factor; Eln, elastin; MMP, matrix metalloproteinases; pax, paired box gene; ngn 3, neurogenin 3; nkx 6.1, NK homeobox factor 6.1; foxa 2, forkhead box A2; pdx 1, pancreatic and duodenal homeobox 1; and MCP-1, monocyte chemoattractant protein 1

MenSCs have been broadly used in preclinical studies, and many of which have shown effectively therapeutic functions in prevention and control of various diseases, including liver disease, diabetes, stroke, Duchenne muscular dystrophy, ovarian-related disease, myocardial infarction, Asherman syndrome, Alzheimer’s disease, acute lung injury, cutaneous wound, endometriosis, and neurodegenerative diseases (Fig. 1). The potential of multi-directional differentiation of MenSCs suggests its potential for repair of various tissue damages. However, the therapeutic effect of MenSCs should not be simply considered as a single reason, we should use a more comprehensive horizon coordinated with the local microenvironment. Especially, some novel hotspots are explored, such as vesicles and exosomes, single-cell RNA-sequencing, and cell-targeted therapy for precision medicine.

In order to achieve the end goal of the use of MenSCs in clinical implementation, the standard criterion of sample collections is needed to produce high quality and high consistency of MenSCs; more importantly, fundamental pre-clinical research is demanded for establishing more treatment strategies and exploring precise signaling pathways. Finally, the long-term safety of MenSCs should be assessed before they are used in clinical medicine. In summary, although more work needs to be done, MenSCs have been proved to play multi-functional roles in treating a variety of diseases through diversely therapeutic strategies in preclinical research, which will be contributed to the development of MenSC-based treatment in regenerative medicine and clinical applications.

## Abbreviations

AD: Adipose tissue
AKT/PKB: Protein kinase B
ALI: Acute lung injury
Angpt: Angiopoietin
Aβ: Amyloid-beta
BALF: Bronchoalveolar lavage fluid
BDNF: Brain-derived neurotrophic factor
BM: Bone marrow
CAD: Coronary artery disease
CCl_4_: Carbon tetrachloride
c-kit: c-kit proto-oncogene
CLI: Critical limb ischemia
c-myc: c-myelocytomatosis
COX-2: Cyclooxygenase-2
CXCR4: C-X-C chemokine receptor type 4
D-Gal: D-galactosamine
DGC: Dystrophy glycoprotein complex
DMD: Duchenne muscular dystrophy
ECM: Extracellular matrix
Eln: Elastin
EMT: Endothelial cell to mesenchymal transition
EOC: Epithelial ovarian cancer
EpCAM: Epithelial cell adhesion molecule
ERK: Extracellular signal-regulated kinases
ET: Endometrial thickness
FET: Frozen embryo transfer
FGF 2: Fibroblast growth factor 2
FHF: Fulminant hepatic failure
foxa 2: Forkhead box A2
FoxO3a: Forkhead O-3a
FOXP3: Forkhead transcription factor-3
GMP: Good manufacturing practice
GRO: Growth-related oncogene
HGF: Hepatocyte growth factor
HIF-1α: Hypoxia inducible factor-1 alpha
HLA-DR: Human leukocyte antigen-DR isotype
HSCs: Hepatic stellate cells
IDO1: Indoleamine 2,3-dioxygenase-1
IFN-γ: Interferon-γ
IL: Interleukin
KGF: Keratinocyte growth factor
LIN: Lineage
LPS: Lipopolysaccharide
MCP-1: Monocyte chemoattractant protein 1
MenSC-Ex: MenSC-derived exosomes
MenSCs: Menstrual blood-derived stem cells
MHC: Major histocompatibility complex
MI: Myocardial infarction
miRs: MicroRNAs
MLR: Mixed lymphocyte reaction
MMP: Matrix metalloproteinases
MSCs: Mesenchymal stem cells
NE: Non-endometriosis
ngn 3: Neurogenin 3
nkx 6.1: NK homeobox factor 6.1
NT-3: Neurotrophin 3
NTFs: Neurofibrillary tangles
OCT-4: Octamer binding transcription factor 4
OGD: Oxygen glucose deprivation
OLT: Orthotopic liver transplantation
OPG: Osteoprotegerin
pax: Paired box gene
PB: Peripheral blood
PBMCs: Peripheral blood mononuclear cells
PCNA: Proliferating cell nuclear antigen
PDGF: Platelet-derived growth factor
pdx 1: Pancreatic and duodenal homeobox 1
POF: Premature ovarian failure
PTEN: Phosphatase and tensin homolog
Sox2: SRY (sex determining region Y)-box 2
SSEA-4: Stage-specific embryonic antigen-4
STAT 3: Signal transducers and activator of transcription 3
STRO-1: Stromal cell antigen 1
T1DM: Type 1 diabetes mellitus
TNF-α: Tumor necrosis factor α
TRA-1-60: Tumor-rejection antigen-1-60
Tregs: Regulatory T cells
UC: Umbilical cords
VEGF: Vascular endothelial growth factor
